# High-Resolution ^1^H NMR Spectroscopy of Fish Muscle, Eggs and Small Whole Fish via Hadamard-Encoded Intermolecular Multiple-Quantum Coherence

**DOI:** 10.1371/journal.pone.0086422

**Published:** 2014-01-17

**Authors:** Honghao Cai, Yushan Chen, Xiaohong Cui, Shuhui Cai, Zhong Chen

**Affiliations:** Department of Electronic Science, Fujian Provincial Key Laboratory of Plasma and Magnetic Resonance, State Key Laboratory of Physical Chemistry of Solid Surfaces, Xiamen University, Xiamen, China; University of Pittsburgh School of Medicine, United States of America

## Abstract

**Background and Purpose:**

Nuclear magnetic resonance (NMR) spectroscopy has become an important technique for tissue studies. Since tissues are in semisolid-state, their high-resolution (HR) spectra cannot be obtained by conventional NMR spectroscopy. Because of this restriction, extraction and high-resolution magic angle spinning (HR MAS) are widely applied for HR NMR spectra of tissues. However, both of the methods are subject to limitations. In this study, the feasibility of HR ^1^H NMR spectroscopy based on intermolecular multiple-quantum coherence (iMQC) technique is explored using fish muscle, fish eggs, and a whole fish as examples.

**Materials and Methods:**

Intact salmon muscle tissues, intact eggs from shishamo smelt and a whole fish (Siamese algae eater) are studied by using conventional 1D one-pulse sequence, Hadamard-encoded iMQC sequence, and HR MAS.

**Results:**

When we use the conventional 1D one-pulse sequence, hardly any useful spectral information can be obtained due to the severe field inhomogeneity. By contrast, HR NMR spectra can be obtained in a short period of time by using the Hadamard-encoded iMQC method without shimming. Most signals from fatty acids and small metabolites can be observed. Compared to HR MAS, the iMQC method is non-invasive, but the resolution and the sensitivity of resulting spectra are not as high as those of HR MAS spectra.

**Conclusion:**

Due to the immunity to field inhomogeneity, the iMQC technique can be a proper supplement to HR MAS, and it provides an alternative for the investigation in cases with field distortions and with samples unsuitable for spinning. The acquisition time of the proposed method is greatly reduced by introduction of the Hadamard-encoded technique, in comparison with that of conventional iMQC method.

## Introduction

Nuclear magnetic resonance (NMR) spectroscopy is a non-invasive, non-destructive, pollution-free and safe experimental technique. Tissue components taken from humans or animals and their percentage variations can be detected by NMR. In areas of disease diagnosis and drug toxicology, detection results provide much useful information [1]–[5]. For liquid samples, NMR spectral information can be obtained through conventional liquid high-resolution (HR) NMR spectroscopy, such as chemical shifts and *J* coupling. However, muscles, tumors, and connective tissues are generally in semisolid phase [6], and may be subject to intrinsic variations in magnetic susceptibility over the sample volume. Caused by this heterogeneity, line broadening, which often cannot be completely overcome by conventional shimming procedures, may lead to peak overlapping and loss of spectral features. Nowadays, there are two traditional methods for HR NMR spectra of tissues: liquid NMR of tissue extracts and HR magic angle spinning (HR MAS) NMR of *ex vivo* tissues. The former has been used for HR NMR spectra of extracts from different tissues [7]–[10]. The information of small metabolites can be obtained from spectra of hydrophilic extracts, while fatty acids distribution can be obtained from spectra of lipophilic extracts. The latter can detect a few milligrams of intact tissues directly without extraction [11]–[14]. The information of both small metabolites and lipids can be obtained simultaneously without any pretreatment. Although these two methods are popular, some limitations exist. For example, complicated pretreatments are required for the extraction method, whereas a specialized Nano rotor or solid-state rotor must be equipped for HR MAS NMR and some vulnerable samples may be damaged due to fast spin. In addition, both methods are unsuitable for *in vivo* applications.

During these years, the method of intermolecular multiple-quantum coherence (iMQC), caused by long-range dipolar interactions among spins in different molecules, has enjoyed wide applications [15]–[19]. Since intermolecular dipolar interactions are effective within a range of 5 ∼ 500 µm, which is much narrower than the size of a typical sample dimension, it is intuitively attractive to apply the iMQC method for HR NMR spectroscopy in inhomogeneous fields [20]. A one-dimensional (1D) HR iMQC NMR spectrum in inhomogeneous magnetic fields requires two-dimensional acquisitions [21], which are time consuming. Recently, Hadamard encoding technique [22] was introduced to iMQC NMR spectroscopy to obtain 1D HR liquid NMR spectra in inhomogeneous magnetic fields [23]. It can provide HR spectra directly and non-invasively through 1D acquisition within a relatively short period of time, even in an extremely strong inhomogeneous magnetic field up to 850 Hz [24], making Hadamard-encoded iMQC a wise choice for investigations of biological tissues.

Salmon is one of the most popular seafoods and possesses great nutritional value. Because both lipids and small metabolites are related to the nourishment and texture of salmon [25], the fish can be classified by quantification of lipids and small metabolites using NMR [8], [11], [26]. The difference between cultured fish and wild fish has caused concerns along with the rapid development of aquaculture [27], [28]. Furthermore, the fish egg is also a certain type of nutritious food. If its component can be identified and quantified, the finding will be of great value to consumers [2], [29], [30].

Salmon muscle tissue is red-and-white interspersed. These two different textures make salmon tissues strongly structured. Although the iMQC spectra of some tissues such as pig brain and brown adipose tissues have been obtained [18], [24], the HR iMQC technique has not been applied to fish studies. In addition, components and structures of salmon muscle tissues are quite different from pig brain tissues and adipose counterparts. The tissue not only includes small metabolites like pig brain tissues, but also contains fats as adipose tissues. So far, there have been NMR studies on fish muscle tissues and fish eggs with tissues extracted [8], [28], [29], [31], [32]. Although HR MAS can detect fish eggs without pretreatments, fragile eggs will be easily destroyed under fast spinning. The technique HR MAS is also powerless for nondestructive detection of the whole fish due to the fact that the sample has to be dissected into small fillets to be fit for the MAS rotor. For the extraction or HR MAS experiments of fish eggs and the whole fish, the cellular structure will be somewhat damaged. In this paper, salmon muscle tissues, shishamo smelt eggs, and intact Siamese algae eater are systematically investigated by the HR Hadamard-encoded iMQC method. The feasibility of HR iMQC spectroscopy is demonstrated through theoretical derivation and experiments. To compare and confirm the results of the iMQC experiment, we also apply HR MAS to same samples.

## Materials and Methods

### Methods

**Figure 1:**
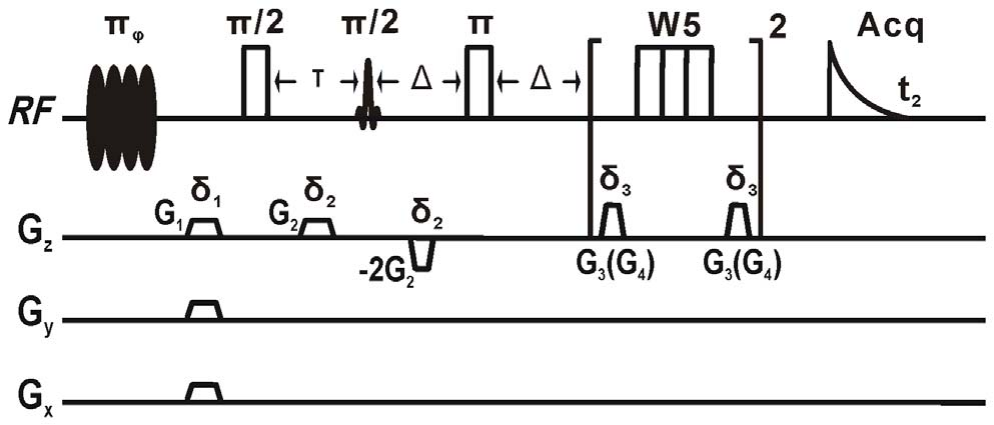
Hadamard-encoded iMQC pulse sequence.

The Hadamard-encoded iMQC pulse sequence is shown in Figure 1. We make a modification on the original sequence [24]: the multiple π pulses is substituted by a single hard π pulse in front of water suppression because signals will decay rapidly when we use multiple π pulses due to the very short relaxation time of fish tissues. At the very beginning of the sequence, a soft polychromatic pulse (π_φ_) [22] only selectively excites solvent spins. Pulse field gradients along *x*, *y* and *z* directions after the soft polychromatic pulse are used to dephase the possible transverse magnetization due to imperfect pulse flipping angle. Two subsequent hard pulses (π/2, π) and a soft pulse (π/2) between them together with the pulse field gradient *G*_2_ are applied to produce the iMQC signal. At last a water suppression module (W5) are introduced to suppress water signal [33].

The iMQC signal expression for the Hadamard-encoded iMQC pulse sequence have been deduced in the previous study [24]. In this paper, a liquid mixture consisting of S, I_1_, and I_2_ components in an inhomogeneous field (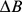) is considered. The component S is an AX spin-1/2 system (including S_k_ and S_l_ spins with a scalar coupling constant *J*_kl_) and I_1_, I_2_ are two isolated spin-1/2 systems, where I_1_ is solvent, and I_2_ and S are solutes. Assume that *ω*_m_ is the frequency offset of spin m (m  =  S_k_, S_l_, I_1_, I_2_) in the rotating frame in the absence of field inhomogeneity.

**Figure 2:**
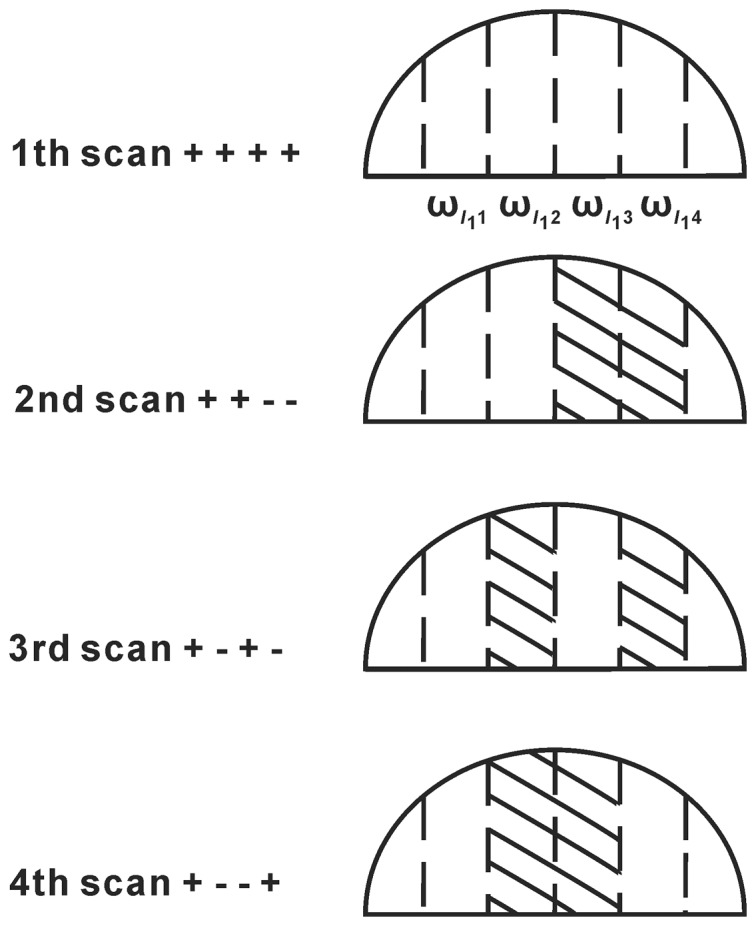
Encoding diagram (Scan times = 4), reproduced according to literature [24].

Since the spectral linewidth of a conventional 1D ^1^H spectrum is greatly increased in an inhomogeneous field, the solvent peak can be divided into N segments (N is the order of Hadamard matrix), and each segment is associated with its own center frequency. These frequencies are encoded according to the Hadamard matrix, and the soft polychromatic pulse in the sequence is generated by this Hadamard matrix. For example, an encoding diagram (N = 4) is shown in Figure 2. The solvent peak is divided into 4 segments, and the 4 center frequencies are 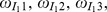 and 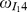, respectively. The 4 frequencies are encoded according to the Hadamard matrix shown in the left column. The linear sign operator *P_i_* (*i*  =  1, 2, 3, 4), which is ‘+’ or ‘−’ in every row of Hadamard matrix represents the effect of Hadamard encoding. The ‘+’ represents that the magnetization is forward, while ‘−’ represents that the magnetization is reversed in the corresponding frequency offset band of solvent spin.

Taking the signal from *S* as an example, we can express its results:
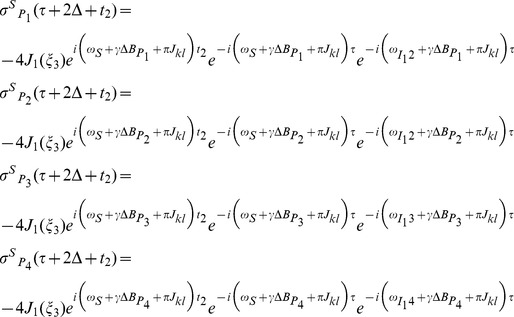
(1)


Where 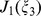 is the Bessel function with integer order *1*; 
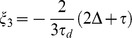
is the argument of the Bessel function; 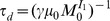 is the dipolar demagnetizing time of *I*_1_ spin, in which *μ*_0_ is vacuum magnetic permeability and *γ* is gyromagenatic ratio, and 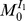
is equilibrium magnetization of *I*_1_ spin. The symbol 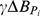 is the frequency offset band of the *i*th component in the polychromatic pulse. From these expressions, we observe that four 1D spectra can be obtained. Each spectrum possesses the same useful spectral information except the chemical shift of signal peaks caused by different solvent excitation frequencies.

### Samples

Fillets of Atlantic salmon (*Salmo salar*) were purchased from the electronic mall and were kept at freezer prior to experiments. The shishamo smelt (*Spirinchus lanceolatus*) was also purchased from the electronic mall and its eggs were surgically harvested from the fish before experiments. The Siamese algae eater (*Crossocheilus siamensis*) was purchased from a local retailer. For iMQC experiments, all samples (salmon muscle tissue, shishamo smelt eggs, and whole Siamese algae eater) were fitted into a 5-mm NMR tube without any pretreatment.

### Data acquisition

All iMQC experiments were performed at 298 K on a Varian NMR System spectrometer with a proton frequency of 499.74 MHz, equipped with a 5-mm ^1^H {^15^N-^31^P} XYZ indirect detection probe with three-dimensional gradient coils. Experiments were performed without field locking, and a four-step phase cycling was applied. The phases for the first selective radio-frequency (RF) pulse and the receiver are (x, y, –x, –y) and (x, x, –x, –x). Hadamard decoding was conducted by using a software package SPROM [34] to obtain HR iMQC spectra. For comparison, 1D HR MAS spin-echo experiments were performed at 298 K on a Bruker AVANCE^III^ spectrometer with a proton frequency of 400.19 MHz equipped with a 4-mm MAS rotor. The sample was spun along the magic-angle (54.7°) direction at a rate of 8 kHz. A *T*_2_-edited CPMG segment with a total echo time of 252 ms was incorporated to selectively record signals of small metabolites. Spectra of 5 kHz spectral width and 32768 data points were obtained using a pulse delay of 5.0 s and a total acquisition time of 13 min. It is necessary to dissect the whole Siamese algae eater into small fillets prior to being placed into the MAS rotor.

**Study of salmon muscle tissue.** For iMQC experiments, an 8×8 Hadamard matrix was used. The duration of the soft polychromatic pulse was 85 ms, and the excitation frequency interval was 20 Hz. It should be noted that the duration of the first soft polychromatic pulse and the order of the Hadamard matrix are determined by the field inhomogeneity and the expected band interval. When the field inhomogeneity becomes large, the rank of the matrix may need to expand. However, the larger this rank expands, experimental results do not necessarily yield HRs. In fact, a Hadamard matrix remains dependent of the sample and the field inhomogeneity. In all experiments, an 8×8 matrix is found to be the best choice. The interval cannot be unlimitedly reduced, as it is inversely proportional to the pulse duration. A smaller interval means a longer pulse duration and, consequently, greater signal attenuation. Therefore, the pulse duration should also be chosen according to the transverse relaxation of the sample. The iMQC parameters were: pulsed field gradients *G*_1_  =  0.1 T/m, *G*_2_  =  0.1 T/m, durations *δ*_1_  =  0.8 ms, *δ*_2_  =  1.0 ms, pulsed field gradients of W5 *G*_3_  =  0.06 T/m, *G*_4_  =  0.30 T/m, durations *δ*_3_  =  1 ms, *δ*_4_  =  1 ms, spectral bandwidth SW =  5 kHz, data points  = 1000, pulse delay  =  4.0 s, echo time 2Δ =  48 ms, and total acquisition time *t*_2_ =  13 min.

**Study of fish eggs.** As mentioned above, for the high field inhomogeneity, it is better to choose an excitation frequency interval that is sufficiently large to fully excite the solvent. Therefore, the duration of the soft polychromatic pulse was 150 ms and the excitation frequency interval was 30 Hz. The iMQC parameters were: pulsed field gradients *G*_1_  =  0.08 T/m, *G*_2_  =  0.09 T/m, durations *δ*_1_  =  0.8 ms, *δ*_2_  =  1.0 ms, pulsed field gradients of W5 *G*_3_  =  0.07 T/m, *G*_4_  =  0.30 T/m, durations *δ*_3_  =  3 ms, *δ*_4_  =  3 ms, spectral bandwidth SW =  5 kHz, data points  =  600, pulse delay  =  4.0 s, echo time 2Δ =  100 ms, and total acquisition time *t*_2_ =  4.5 min. Since fish eggs are fragile, magnified photos of the eggs before and after MAS were taken to see the morphologic changes under rotation by a Huaqi DLC300 electronic microscope.

**Study of the whole fish.** We chose a whole Siamese algae eater with the appropriate size for the 5-mm NMR tube to further test the feasibility of the HR iMQC method for intact organisms with intrinsic macroscopic susceptibility gradients. Prior to the iMQC experiment, the spin echo MR images of its sagittal and axial planes were acquired on the Varian NMR System spectrometer with TR/TE  =  2000/22 ms, and image matrix  =  512×512 in circa 17 min. The duration of the soft polychromatic pulse was 150 ms and the excitation frequency interval was 30 Hz. The iMQC parameters included: pulsed field gradients *G*_1_  =  0.08 T/m, *G*_2_  =  0.09 T/m, durations *δ*_1_  =  0.8 ms, *δ*_2_  =  1.0 ms, pulsed field gradients of W5 *G*_3_  =  0.07 T/m, *G*_4_  =  0.30 T/m, durations *δ*_3_  =  3 ms, *δ*_4_  =  3 ms, spectral bandwidth SW =  5 kHz, data points  =  600, pulse delay  =  4.0 s, echo time 2Δ =  48 ms, and total acquisition time *t*_2_ =  18 min.

## Results

**Figure 3:**
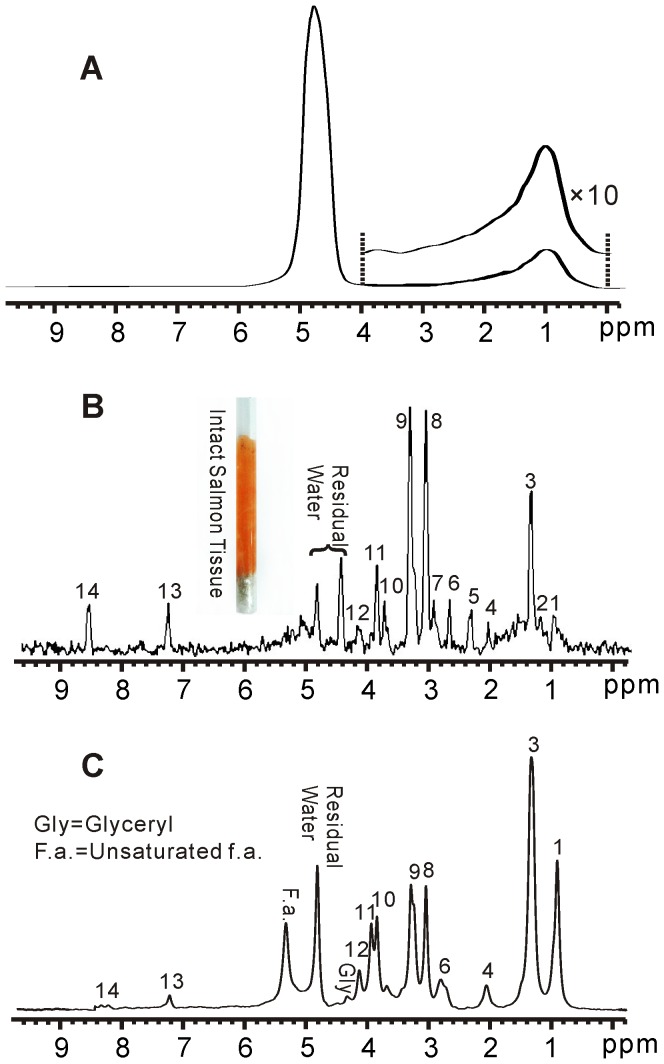
^1^H NMR spectra of intact muscle tissue from Atlantic salmon. (A) Conventional 1D spectrum. (B) 1D iMQC spectrum. (C) 1D HR MAS spectrum.

The *ex vivo* ^1^H NMR spectra of intact muscle tissue from Atlantic salmon are shown in Figure 3. The conventional 1D spectrum is shown in Figure 3A. Because of the intense and broad water signal and inhomogeneous broadening resulted from magnetic susceptibility gradients among the solid/half-solid biological tissue, hardly any spectral information can be obtained (Figure 3A). The Hadamard-encoded iMQC 1D spectrum is acquired under the same circumstance. The water signal is effectively suppressed by W5 solvent suppression module. An 8×8 Hadamard matrix is used and the excitation frequency interval is 20 Hz. After 8 scans, a HR spectrum can be decoded (Figure 3B). The information of chemical shifts is maintained. For comparison, an HR MAS spectrum is shown in Figure 3C. According to the MAS spectrum and the literature [35], the assignment of the observed signals in the iMQC spectrum of intact muscle tissue is given in Table 1. It can be observed that most of fatty acids as well as small metabolites are present in the iMQC spectrum except glyceryl (4.30 ppm) and unsaturated fatty acids (5.34 ppm).

**Table 1:** Peak assignment for iMQC spectrum of intact muscle tissue from Atlantic salmon.

Peak No.	Compound	Proton (s)	Chemical Shift (ppm)
1	All f.a. except n-3 f.a.	-CH_3_	0.92
2	Unassigned	-CH_3_	1.20
3	All f.a. except 20:5 and 22:6	-(CH_2_)_n_-	1.37
4	Unsaturated f.a.	-C**H**_2_-CH = CH	2.02
5	All f.a. except 22:6	-CH_2_-COOR	2.32
6	Polyunsaturated f.a.	= CH-C**H**_2_-CH =	2.70
7	Anserine	-CH_2_	2.90
8	Creatine	-N-CH_3_	3.05
9	Choline/Anserine	-N-(CH_3_)_3_/-CH_2_	3.20∼3.30
10	Anserine	-N-CH_3_	3.75
11	Creatine	-CH_2_	3.90
12	Lactate	-CH	4.16
13	Histidine	-CH	7.23
14	Histidine	-CH	8.56

Chemical shifts are referenced to the water signal (4.80 ppm), f.a.  =  fatty acids.

**Figure 4:**
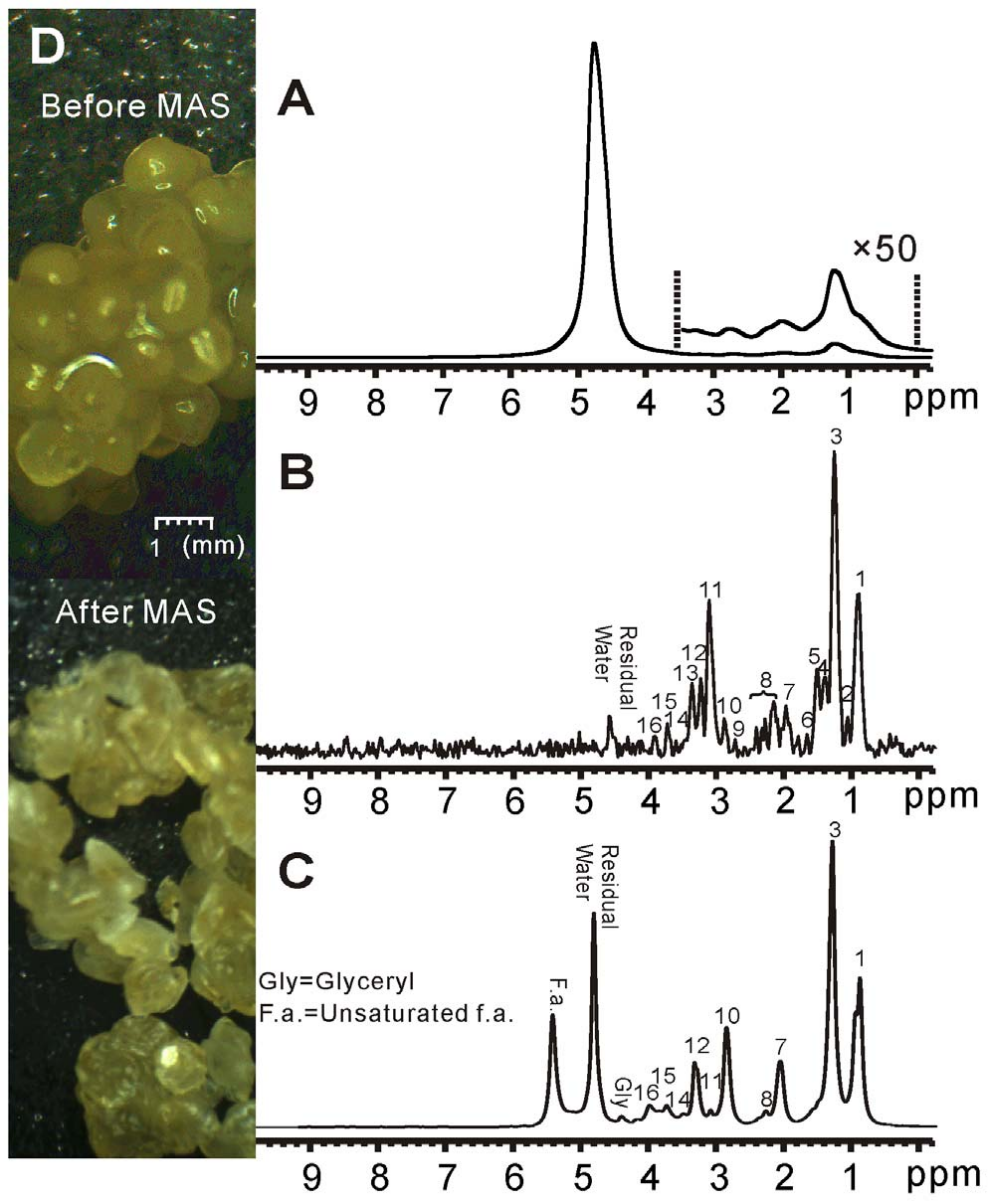
^1^H NMR spectra of shishamo smelt eggs. (A) Conventional 1D spectrum. (B) 1D iMQC spectrum. (C) 1D HR MAS spectrum. (D) Fifty-time magnified photos of eggs before and after MAS.

The results of shishamo smelt eggs are shown in Figure 4. Likewise, hardly any spectral information can be obtained from the conventional 1D spectrum due to inhomogeneous line broadening (Figure 4A). After Hadamard-encoded iMQC 1D acquisition and spectrum decoding, an HR spectrum can be obtained (Figure 4B). An HR MAS spectrum of intact shishamo smelt eggs is also obtained, as shown in Figure 4C. According to the MAS spectrum and the literature [35], the assignment of observed signals in the iMQC spectrum of intact shishamo smelt eggs is provided in Table 2. Similar to the iMQC spectrum of salmon muscle tissue, most of signals from small metabolites and fatty acids are recovered except glyceryl (4.40 ppm) and unsaturated fatty acids (5.40 ppm). However, the signal intensity of fatty acids relative to small metabolites in shishamo smelt eggs in Figure 4B is much higher than that in salmon muscle tissue in Figure 3B. This phenomenon coincides with previous reports [36]–[38]. The MAS experiments give the same results (Figure 3C and 4C). Photos of fish eggs, which are taken by electronic microscope prior to and after the MAS experiment with 50-time magnified are shown in Figure 4D. As can be seen, the eggs, which are globular and plump prior to MAS, become shriveled and bleached. This phenomenon may be related to the rupture of egg membranes and the dehydration of egg cells under high-speed rotation.

**Table 2:** Peak assignment for iMQC spectrum of shishamo smelt eggs.

Peak No.	Compound	Proton (s)	Chemical Shift (ppm)
1	All f.a. except n-3 f.a.	-CH_3_	0.90
2	n-3 f.a.	-CH_3_	1.05
3	All f.a. except 20:5 and 22:6	-(CH_2_)_n_-	1.37
4	Lactate	-CH	1.40
5	All f.a. except 22:6	-C**H**_2_-CH_2_-COOR	1.55
6	20:5 f.a	-C**H**_2_-CH_2_-COOR	1.65
7	Unsaturated f.a.	-C**H**_2_-CH = CH	2.00
8	Glutamate/Glutamine	-CH/-CH_2_	2.10∼2.40
9	Polyunsaturated f.a.	= CH-C**H**_2_-CH =	2.73
10	Anserine	-CH_2_	2.90
11	Creatine	-N-CH_3_	3.08
12	Choline	-N-(CH_3_)_3_	3.22
13	Anserine	-N-CH_3_	3.31
14	Glycine	-CH_2_	3.60
15	Anserine	-N-CH_3_	3.75
16	Creatine	-N-CH_2_-	3.93

Chemical shifts are referenced to the water signal (4.80 ppm), f.a.  =  fatty acids.

**Figure 5:**
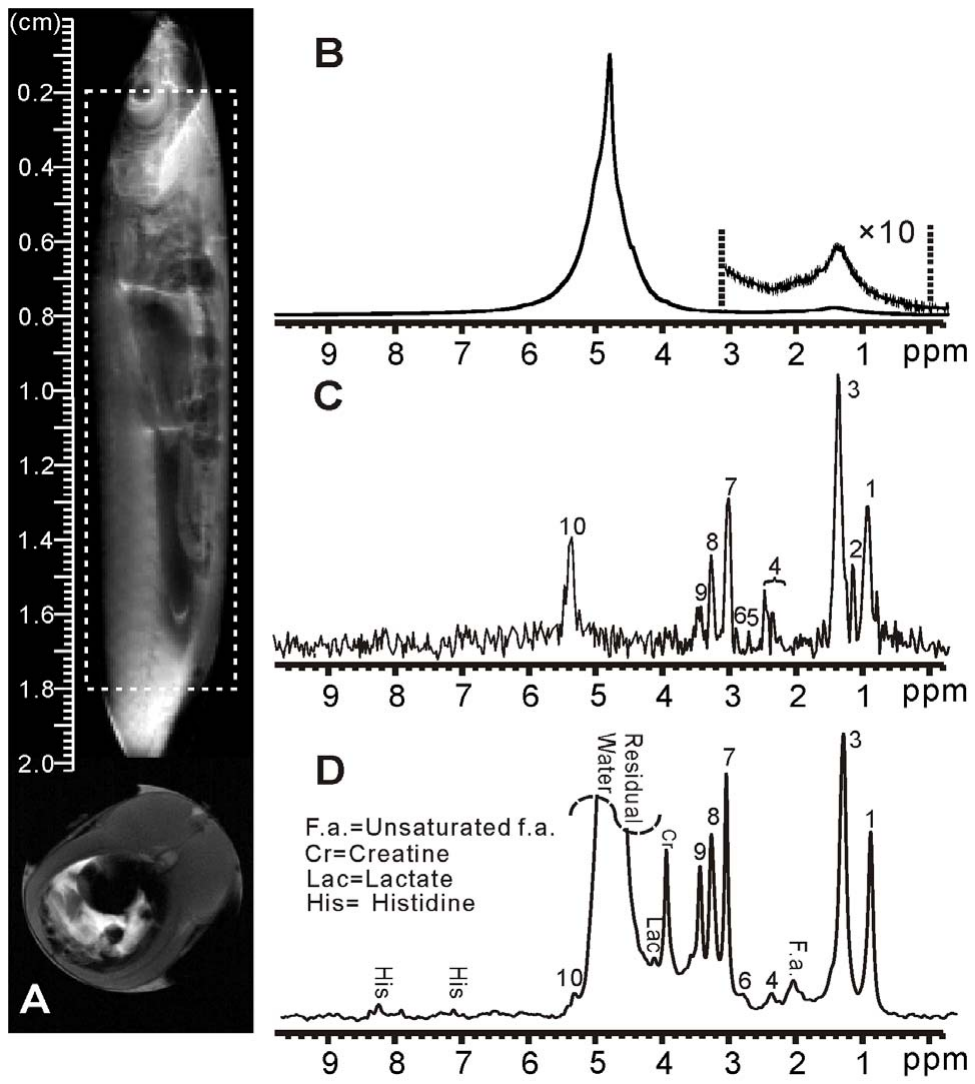
^1^H NMR spectra of intact Siamese algae eater. (A) Sagittal and axial spin-echo images. (B) Conventional 1D spectrum. (C) 1D iMQC spectrum. (D) 1D HR MAS spectrum.

Figure 5 shows the results of the whole Siamese algae eater. The location of the fish in the NMR tube is displayed by the anatomic spin-echo images along sagittal and axial orientations in Figure 5A. From these images, we can find that the whole fish spans about 20 mm, covering the detection area denoted by a dashed box whose length is 16 mm. The conventional 1D spectrum is shown in Figure 5B. From the spectrum, it can be found that there exists only a large water signal due to the pronounced field inhomogeneity. The iMQC spectrum and HR MAS spectrum are also shown in Figures 5C and D. Small metabolites and fatty acids are observable. However, compared to the HR MAS spectrum, some signals disappear in iMQC spectrum such as unsaturated fatty acids (2.03 ppm), creatine (3.95 ppm), lactate (4.14 ppm) and histidine (7.13 ppm, 8.12 ppm). According to the MAS spectrum and the literature [35], the assignment of the observed signals in the iMQC spectrum of the whole Siamese algae eater is shown in Table 3.

**Table 3:** Peak assignment for iMQC spectrum of intact Siamese algae eater.

Peak No.	Compound	Proton (s)	Chemical Shift (ppm)
1	All f.a. except n-3 f.a.	-CH_3_	0.90
2	n-3 f.a.	-CH_3_	1.10
3	All f.a. except 20:5 and 22:6	-(CH_2_)_n_-	1.37
4	Glutamate/Glutamine	-CH/-CH_2_	2.20∼2.45
5	Polyunsaturated f.a.	= CH-C**H**_2_-CH =	2.73
6	Anserine	-CH_2_	2.90
7	Creatine	-N-CH_3_	3.05
8	Anserine	-N-CH_3_	3.28
9	Taurine	-S-CH_2_	3.45
10	Unsaturated f.a.	-N-CH_3_	5.38

Chemical shifts are referenced to the water signal (4.80 ppm), f.a.  =  fatty acids.

To quantitatively and specifically understand the performance of the iMQC method on these three fish samples relative to the HR MAS, we calculate and list the spectra-related information such as the sensitivity and the linewidth of resonances in Table 4 based on the spectra of three samples (Figures 3, 4 and 5) and the spectral assignments (Tables 1, 2 and 3). The sensitivity is calculated as 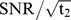, where *t* ^­^_2­­_  =  acquisition time [39], and SNR is the signal to noise ratio calculated by dividing the intensity of the creatine peak (3.05 ppm) by the standard deviation (SD) of noises in the region between 6.0 and 6.5 ppm. The linewidth at half maximum of creatine peak (3.05 ppm) as a representative is measured. For the sample weight, only the detection area is taken into account, and the total acquisition time is also presented.

**Table 4:** Comparison of spectra-related information from the iMQC and MAS spectra*.

Sample	Sensitivity**	Linewidth*** (Hz)	Sample weight (mg)	Total acquisition time (min)
Salmon muscle tissue	26.7/63.5	27.2/25.9	223.53/111.02	9/13
Eggs of shishamo smelt	23.5/119	40.0/30.4	242.27/113.03	4.5/13
Whole Siamese algae eater	6.04/53.0	44.2/23.5	216.80/123.26	18/13

The left and right values are corresponding to the results of iMQC and MAS, respectively.

The sensitivity is calculated as 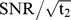, where *t*_2_  =  acquisition time, and SNR is the signal to noise ratio calculated by dividing the intensity of the creatine peak (3.05 ppm) by the standard deviation of noises in the region between 6.0 and 6.5 ppm.

The linewidth at the half maximum of creatine peak (3.05 ppm) as a representative is measured.

## Discussion

From experimental results, we find that, compared to HR MAS, most of fatty acids as well as the small metabolites are conspicuous in the iMQC spectra. Nevertheless, some resonances have vanished in the proposed experiment, such as glyceryl (4.30 ppm) and unsaturated fatty acids (5.34 ppm) which appear in MAS spectra of salmon muscle tissues and shishamo smelt eggs. This situation may be caused by the side effect of the solvent suppression since these two resonances are adjacent to the large water peak. However, for the iMQC spectrum of the whole Siamese algae eater (Figure 5C), other peaks, such as fatty acids (2.03 ppm), creatine (3.95 ppm), lactate (4.14 ppm) and histidine (7.13 and 8.12 ppm), have disappeared. This result may attributed due to the more complex circumstance, induced by magnetic susceptibility variation between the fish and the air in the interspaces in NMR tube and the intrinsic magnetic susceptibility variation in fish itself among muscle tissues, bones, viscera and fish scale. The low concentration of some small metabolites poses another possible reason. Furthermore, signals of creatine (3.95 ppm) and lactate (4.14 ppm), which are adjacent to the intense water peak, may also be affected by the solvent suppression. The assigned peaks of these three samples (Tables 1, 2 and 3) are similar. A few peaks, which appear in one sample’s spectrum but are absent in other samples’ spectra, may be attributed to the inhomogeneity of different extents and the diverse content of components from various samples. Furthermore, it has been found that the relative peak intensity of the iMQC spectra is unlike that of the MAS spectra. The difference may arise from the transverse relaxation decay in different degrees determined by different spin echo time and pulse field gradients used in iMQC and MAS experiments.

Aside from the comparison of resonances in the iMQC and MAS spectra, the spectra-related information is also analyzed. It can be found that sensitivities of the iMQC signals of salmon muscle tissues, shishamo smelt eggs and the whole Siamese algae eater are only about 42%, 20%, and 11% of those of HR MAS signals while the sample weight for the iMQC is half that for MAS. These results are attributed mainly to the intrinsic low SNR of iMQC signals. Moreover, compared to that of the salmon muscle tissue and shishamo smelt eggs, the iMQC sensitivity of the whole Siamese algae eater is dramatically decreased. Such a status may be related to the more serious field inhomogeneity originated from more complex structures of the whole organism and the magnetic susceptibility variation between the fish and the air in the interspaces mentioned above. It is worth noting that the MAS sensitivity of shishamo smelt eggs is higher than that of salmon muscle tissue due to the reason that shishamo smelt eggs tends to be in liquid state during rotation. On the contrary, the iMQC sensitivity of shishamo smelt eggs is unexpectedly lower than that of salmon muscle tissue. It may be related to the signal decay caused by the inhomogeneity originated from the uneven filling of shishamo smelt eggs in NMR tube, because shishamo smelt eggs are too tender to be filled uniformly.

For shishamo smelt eggs and whole Siamese algae eater, linewidths of creatine in the iMQC spectra are broader than those in the MAS spectra, while for the salmon muscle tissue, the linewidth of creatine in the iMQC spectrum is close to that in the MAS spectrum. Theoretically, the optimal spectral linewidth is close to the excitation frequency interval, which is 20, 30, and 30 Hz for salmon muscle tissues, shishamo smelt eggs, and whole Siamese algae eater, respectively. The experimental results basically agree with the theoretical values. As pointed above, the resolution of decoded spectrum mainly depends on the excitation frequency interval. When the field inhomogeneity increases, the resolution is also affected by the signal attenuation caused by *T*_2_ relaxation and diffusion. Linewidths of fish eggs and whole fish are much broader than those of the muscle tissue. On one hand, they depend on the excitation frequency interval, which is 30 Hz for both shishamo smelt eggs and whole Siamese algae eater, larger than that of salmon muscle tissues (20 Hz). On the other hand, this difference in linewidths is due to the intrinsic short relaxation times caused by the inhomogeneity in fish eggs and the whole fish, as discussed above.

All experimental results show that HR NMR spectra of the *ex vivo* salmon muscle tissue, *ex vivo* shishamo smelt eggs and intact whole Siamese algae eater can be obtained by the Hadamard-encoded iMQC method. Along with the W5 solvent suppression technique, resolution-enhanced spectra can be recovered, and the chemical shift information can be retained. Although the resolution and the sensitivity of iMQC spectrum are not as high as those of MAS spectrum due to limitations of the Hadamard-encoded technique and the iMQC method, the proposed method requires no pretreatments and is suitable for non-invasively investigation. These merits make the iMQC method a promising tool in the cases with field distortions, such as the studies of tumors, after-surgery tissues, or whole organs. Compared to the conventional iMQC techniques by which the acquisition of a HR spectrum can be accomplished [21], [40], the Hadamard-encoded technique greatly reduces the acquisition time.

## Conclusions

In this work, a Hadamard-encoded iMQC technique is used to obtain HR 1D ^1^H NMR spectra of fish muscles, fish eggs, and the whole fish. Experimental results verify the feasibility of the technique in detecting structured semisolid-state samples comprised of complex components and even a whole organism. Other than HR MAS, the iMQC technique provides an alternative approach for HR semisolid-state NMR spectroscopy. Although the sensitivity of iMQC is not as high as that of HR MAS, the iMQC method remains advantageous. It can be used in non-invasive detection and does not require any pretreatment, a particular Nano rotor, and a solid-state rotor. For these reasons, the iMQC method can be a proper complement to HR MAS when samples are unsuitable for spinning. Moreover, it is capable of shortening the shimming time and the acquisition time. The lower intensity of iMQC signals relative to conventional SQC signals is the main obstacle for practical applications. Stronger magnetic fields, more sensitive coils, and dynamic nuclear polarization technique are possible ways to overcome this problem [41], [42], and belongs to the scope of the future work.
